# Inzidenz und Beginn schwerer kardialer Ereignisse nach der Strahlentherapie von Ösophaguskarzinomen

**DOI:** 10.1007/s00066-020-01693-x

**Published:** 2020-09-30

**Authors:** Mechthild Krause

**Affiliations:** Klinik und Poliklinik für Strahlentherapie und Radioonkologie, Universitätsklinikum Carl Gustav Carus an der Technischen Universität Dresden, Anstalt des öffentlichen Rechts des Freistaates Sachsen, Fetscherstraße 74, 01307 Dresden, Deutschland

## Ziel der Arbeit

Das Auftreten einer späten Kardiotoxizität im Zusammenhang mit Strahlentherapie bei Brustkrebs und Hodgkin-Lymphom ist gut bekannt. Die relativ hohe kardiale Dosisexposition bei der Radiotherapie von Ösophaguskarzinomen kann zu einem früheren Ausbruch von Herzerkrankungen führen. Für diese Publikation wurden die Inzidenz, der Beginn und die Langzeitüberlebensresultate von hochgradigen kardialen Ereignissen untersucht, die nach Strahlentherapie in einer großen Kohorte von Patienten mit Ösophaguskarzinom auftraten [2].

## Patientengut und Methode

Zwischen März 2005 und August 2017 wurden 479 Patienten mit Ösophaguskarzinom aus einer prospektiven institutionellen Datenbank am MD Anderson Cancer Center in Houston analysiert. Alle Patienten wurden entweder mit intensitätsmodulierter Strahlentherapie (IMRT, *n* = 320) oder Protonentherapie (PBT, *n* = 159) behandelt. Die Strahlentherapie erfolgte entweder präoperativ oder definitiv. Ausgewertet wurde jedes Grad-3(G3)- oder höhergradige (G3+) kardiale Ereignis gemäß CTCAE, Version 5.0.

## Ergebnisse

kardiale G3+-Ereignisse traten bei 18 % der Patienten im Median nach 7 Monaten auf bei einer medianen Nachbeobachtungszeit von 76 Monaten. Vorbestehende Herzerkrankung (*P* = 0,001) und Bestrahlungsmodalität (IMRT vs. PBT; *p* 0,027) waren signifikant mit kardialen G3+-Ereignissen assoziiert. In der multivariaten Analyse war die mittlere Herzdosis, insbesondere <15 Gy, mit reduzierten Ereignissen G3+ assoziiert. Darüber hinaus waren Herzereignisse G3+ mit einem schlechteren Gesamtüberleben verbunden.

## Schlussfolgerungen der Autoren

Schwere kardiale Ereignisse waren relativ häufig mit einem frühen Ausbruch bei Ösophaguskarzinompatienten nach Strahlentherapie assoziiert, insbesondere bei Patienten mit bereits bestehenden Herzerkrankungen und höheren Strahlendosen im Herzen. Es sollten optimale Behandlungsansätze gewählt werden, um die kumulativen Dosen auf das Herz, insbesondere bei Patienten mit vorbestehender Herzerkrankung, zu reduzieren.

## Kommentar

Kürzlich wurde die erste randomisierte Studie publiziert, die Protonen vs. Photonen (IMRT) bei definitiv oder neoadjuvant mit Radiochemotherapie behandelten Patienten mit Ösophaguskarzinom miteinander verglich. Hier zeigte sich ein Vorteil der Protonentherapie in den kumulativen gewichteten Toxizitäten und postoperativen Komplikationen [1], der überwiegend auf die Reduktion der postoperativen kardialen und pulmonalen Komplikationen nach der Protonentherapie zurückzuführen zu sein schien. Eine randomisierte multizentrische Phase-III-Studie zu dieser Fragestellung läuft derzeit. Dieselbe Arbeitsgruppe hat nun aus einer großen institutionellen prospektiven Datenbank nochmals gezielt die schweren kardialen Toxizitäten nach Protonentherapie mit denen nach IMRT verglichen [2]. Auch hier wurden primäre oder neoadjuvante Radiochemotherapien einbezogen, zum Teil mit Induktionschemotherapie. Die Strahlendosen lagen zwischen 41,4 und 50,4 Gy. Der primäre Endpunkt, kardiale Ereignisse G3+, wurde nach CTCAE klassifiziert und enthielt Arrhythmien, Herzstillstand, Herzinsuffizienz, akute Koronarereignisse, Perikarditis und Perikarderguss. Der auswertende Arzt war gegenüber dem Bestrahlungsplan verblindet, die ausgewerteten G3+-Ereignisse wurden unabhängig durch 3 Kardiologen anhand der Unterlagen verifiziert. Sekundärer Endpunkt war das Gesamtüberleben der Patienten.

Insgesamt 23 % der Patienten hatten vorbestehende Herzerkrankungen. Bei einer mittleren Nachbeobachtungszeit von 76 Monaten traten bei 18 % der Patienten kardiale Ereignisse G3+ auf. Die Zeit bis zum kardialen Ereignis betrug im Median 7 Monate, 81 % der Ereignisse traten innerhalb von 2 Jahren nach der Radiochemotherapie auf. Patienten mit vorbestehender Herzerkrankung jeglicher Art hatten ein signifikant höheres Risiko für ein kardiales Ereignis G3+ nach der Radiochemotherapie (2-Jahres-Raten 22 % vs. 13 %; 5‑Jahres-Raten 24 % vs. 16 %; *p* = 0,006), während die Durchführung der Operation nach der Radiochemotherapie nicht zu einer signifikanten Risikoerhöhung führte. Patienten nach IMRT hatten ein signifikant höheres Risiko, ein kardiales Ereignis G3+ zu erleiden, im Vergleich zu Patienten nach Protonentherapie (Hazard ratio [HR] = 1,746; 95 %-CI 1,065–2,862; *P* = 0,027). Die Herzdosis war signifikant höher bei IMRT im Vergleich zur Protonentherapie: V5Gy (Volumen in % das mindestens 5 Gy erhält) (IMRT vs. PBT; 87,09 ± 20,16 vs. 41,65 ± 18,67; *P* = 0,000), V30Gy (IMRT vs. PBT; 27,46 ± 16,69 vs. 19,33 ± 8,67; *P* = 0,000) und mittlere Herzdosis (IMRT vs. PBT; 21,55 ± 7,01 Gy vs. 12,50 ± 4,97 Gy; *P* = 0,000). Die beiden Patientengruppen waren ausgeglichen zusammengesetzt mit der Ausnahme, dass die Protonenpatienten im Durchschnitt älter waren und häufiger kardiale Vorerkrankungen aufwiesen. In einer Subgruppenanalyse hatten Patienten mit vorbestehenden Herzerkrankungen einen größeren Vorteil durch die Protonentherapie im Sinne einer Reduktion der kardialen Ereignisse G3+ (2-Jahres-Raten 30 % vs. 11 %; 5‑Jahres-Raten 32 % vs. 14 %; *P* = 0,018) verglichen mit der Gruppe ohne Herzvorerkrankungen (2-Jahres-Raten 14 % vs. 11 %; 5‑Jahres-Raten 18 % vs. 13 %; *P* = 0,345). In der Analyse der dosimetrischen Daten waren univariat die V5Gy und die mittlere Herzdosis, multivariat die mittlere Herzdosis signifikant mit G3+ kardialen Ereignissen vergesellschaftet (HR = 1,034; 95 %CI, 1,006–1,062; *p* = 0,015). Für das Gesamtüberleben waren multivariat das klinische Stadium der Tumorerkrankung (III vs. I/II, HR = 1,789; 95 %-CI 1,354–2,364; *p* = 0,000), durchgeführte Operation (ja vs. nein, HR = 0,519; 95 %-CI 0,405–0,665; *p* = 0,000) und kardiale Ereignisse G3+ (ja vs. nein, HR = 1,363; 95 %-CI 1,013–1,834; *P* = 0,041) signifikante prognostische Faktoren. Das Gesamtüberleben nach 2 Jahren und nach 5 Jahren für Patienten mit vs. ohne kardiales Ereignis G3+ war 38 % vs. 52 % und 59 % vs. 74 %.

Bei einer Tumorart, in der ähnlich wie beim Lungenkarzinom ein hoher Prozentsatz der Patienten eine oder mehrere kardiale Vorerkrankungen aufweist, haben diese Daten eine sehr große Bedeutung. Sie zeigen ähnlich wie beim Lungenkarzinom [3] ein frühes Auftreten der kardialen Ereignisse innerhalb der ersten 2 Jahre nach der Radiochemotherapie. Diese Daten untermauern die Notwendigkeit eines kardialen Screenings von Patienten, die im Thoraxbereich bestrahlt werden. Patienten mit vorbestehenden Herzerkrankungen und hoher Herzdosis, wie sie bei der Strahlentherapie des Ösophaguskarzinoms meist bestehen, sollte eine Protonentherapie angeboten werden. Diese Patienten sollten auch in der Nachsorge kardiologisch mitbetreut werden. Bisher gibt es keine einheitlichen Leitlinien zu Dosis-Constraints für das Herz bei Bestrahlung von Ösophagus- oder Lungenkarzinomen. Die NCCN empfiehlt, die mittlere Herzdosis bei <30 Gy zu halten. Dies erscheint anhand der vorliegenden Daten zu hoch. Die Autoren der hier kommentierten Arbeit empfehlen, eine mittlere Herzdosis von maximal 15 Gy einzuhalten.

## Fazit

Es handelt sich um eine der größten Studien zu den schweren kardialen Ereignissen nach Strahlentherapie des Ösophaguskarzinoms und die erste, die einen Vergleich zwischen der Strahlentherapie mit Protonen und denen nach IMRT anstellt. Trotz der retrospektiven Betrachtung ist die Qualität hoch. Wesentliche Aussage: Die Herzdosis sollte bei thorakalen Bestrahlungen wegen Ösophaguskarzinom deutlich reduziert werden, laut Empfehlung der Autoren auf eine mittlere Herzdosis von <15 Gy. Insbesondere Patienten mit kardialen Vorerkrankungen sollte eine Protonentherapie angeboten werden, um die Herzdosis weitestgehend zu senken. Inwiefern kardiale Interventionen das Risiko von Patienten mit Vorerkrankungen wieder reduzieren können, ist bisher ungeklärt.
